# Direct Oral Anticoagulants and Timing of Hip Fracture Surgery

**DOI:** 10.3390/jcm9072200

**Published:** 2020-07-12

**Authors:** Seth M. Tarrant, Michael J. Catanach, Mahsa Sarrami, Matthew Clapham, John Attia, Zsolt J. Balogh

**Affiliations:** 1 John Hunter Hospital, Lookout Rd, New Lambton Heights, NSW 2305, Australia; seth.tarrant@uon.edu.au (S.M.T.); michael.catanach@health.nsw.gov.au (M.J.C.); 2 University of Newcastle, Callaghan, NSW 2308, Australia; mahsa.sarrami@health.nsw.gov.au (M.S.); John.Attia@newcastle.edu.au (J.A.); 3 Hunter Medical Research Institute, New Lambton Heights, NSW 2305, Australia; Matthew.Clapham@hmri.org.au

**Keywords:** hip fracture, DOAC, NOAC, anticoagulant, antithrombotics, mortality, outcomes

## Abstract

Timely surgical intervention in hip fracture has been linked to improved outcomes. Direct Oral Anticoagulants (DOACs) are an emerging class of anticoagulants without evidence-based guidelines on surgical timing. This study aims to investigate how DOACs affect surgical timing and hence perioperative outcomes. A retrospective database/registry review was conducted for geriatric hip fracture patients aged 65 and over between 2011 and 2018. Primary outcome was 30-day mortality. Secondary outcomes included serious adverse events (SAE), transfusion and postoperative day (POD) 1 haemoglobin (Hb) levels. From a cohort of 3264 patients, 112 admitted subjects were taking DOACs; the annual proportion on DOACs increased over time. Mean time to surgery from last dose (T_s_) was 2.2 (±1.0 SD) days. The primary outcome, 30-day mortality, occurred in 16 (14%) patients with secondary outcomes of SAEs in 25 (22%) patients and transfusion in 30 (27%) patients. T_s_ (days) did not significantly affect 30-day mortality (odds ratio (OR): 1.37, 95% confidence interval (CI): 0.80–2.33; *p* = 0.248), SAE (hazard ratio (HR): 1.03, 95% CI: 0.70–1.52; *p* = 0.885), transfusion (OR: 0.72 95% CI: 0.45 to 1.16; *p* = 0.177) or POD 1 Hb (OR: 1.99, 95% CI: −0.59 to 4.57; *p* = 0.129). Timing of surgery does not influence common surgical outcomes such as 30-day mortality, SAE, transfusion, and POD1 Hb in patients taking DOACs on admission.

## 1. Introduction

Cardiac and vascular comorbidities are common in patients who sustain hip fracture [1]. Consequently, many patients are taking blood thinners upon admission; antithrombotic usage has been steadily increasing over the last two decades [2], reaching approximately 50% in first world populations [3]. The management of antithrombotics in the face of surgery is a perennial question: cessation of blood thinners is of concern with immobility-related thromboembolic complications and the loss of protective effects leading to perioperative complications [4], but on the other hand, early intervention carries the perceived risk of surgical bleeding and the need for postoperative transfusion.

The use of Direct Oral Anticoagulants (DOACs) has increased within the geriatric population. National pharmaceutical subsidies have resulted in a steady increase in usage [5]. The existing literature examining DOAC use in hip fracture is small [6,7,8,9,10,11,12,13,14,15,16], and often focuses on comparing DOAC cohorts with groups not taking them, which results in differences between group comorbidity profiles. 

The appropriate perioperative management for DOACs in hip fracture has been recently queried [17], with expert opinion suggesting a waiting period of 24–48 hours, based on DOAC half-lives and guidelines for elective surgery [18]. Factor Xa levels are possible to perform with patients taking apixaban, rivaroxaban and edoxaban, but how they relate to surgical timing in hip fracture unknown. Several options are available for reversal, but they are expensive and seldom utilised given the subacute nature of hip fracture surgery [18,19].

This study aims to explore the optimal surgical timing of hip fracture patients only taking DOACs, with transfusion, morbidity and mortality as key short term outcomes associated with potential increased perioperative blood loss. We hypothesise that surgical timing will not affect these outcomes.

## 2. Patients and Methods

### 2.1. Study Setting

An observational study was undertaken from patients in a University-affiliated Level 1 Trauma centre that treats approximately 400 geriatric hip fractures annually. Comanagement with orthogeriatric shared care has occurred since 2014. Operative timing is generally surgeon-driven, with the majority of surgery occurring on the day of or the day after admission, depending on operating room availability and the burden of high-energy trauma; the institutional median time to surgery for hip fracture is 23 hrs [20]. The study had ethical approval at the Hunter New England Human Research Ethics Committee (AU201803-13). 

### 2.2. Population

Patients were consecutively entered into the institutional Long Bone Fracture Database from 2010 to 2014 and the National Hip Fracture Registry between 2015 and December 2018. All database records were retrospectively reviewed regarding admission on a DOAC. Inclusion criteria were patients aged 65 or over, admitted with a proximal femur fragility fracture, and taking a DOAC. Exclusion criteria from the hip fracture database and registry were a mechanism that was not low-energy, pathological fracture, and fracture that involved a prosthesis. Patients that had not taken a DOAC dose within 48 hours of admission were excluded from analysis. A control group not taking antithrombotic medication was matched using age, sex and year of admission, for comparison of institutional performance.

### 2.3. Data

Demographic, perioperative and outcome details were collected. Age, sex, residence, mobility status and cognitive status were collected. Charlson Comorbidity Index (CCI) (nonage adjusted) was collected as a surrogate marker of comorbidity [21]. Fracture type was graded by the Muller classification [22] by an Orthopaedic surgeon. The type of DOAC, dosing regimen, indication for drug and last dose were recorded routinely. Drug levels and/or Factor Xa levels were not universally ordered in our institution and therefore not included in analysis. As all patients were pre-injury prescribed DOACs, it was assumed that anticoagulation was at an adequately therapeutic level. Time to surgery after last DOAC dose (T_s_), type of operation, type of anaesthesia and any reason for operative delay were documented. Perioperative haemoglobin (Hb) and admission estimated glomerular filtration rate (eGFR) were recorded.

### 2.4. Outcomes

Primary outcome was 30-day mortality. Secondary outcomes were the number of packed red cells transfused, postoperative day (POD) 1 Hb, and time to serious adverse events (SAE) as defined by Menendez and Ring [23], combined with sudden death if no SAE was identified. Mortality was derived from the National Hip Fracture Registry and Registry of Births, Deaths and Marriages. Cause of death was derived from doctor-certified hospital records. Strict perioperative transfusion criteria do not exist in our centre; however, patients with haemoglobin concentrations below 70 g/L will generally be transfused. Haemoglobin concentrations between 70 and 100 g/L with dyspnoea, asthenia or a history of ischaemic heart disease; chronic renal failure; or chronic airway limitation will generally be transfused. Intraoperative transfusion is highly dependent on haemodynamic physiology, surgical blood loss and arterial blood gas parameters.

SAEs included myocardial infarction, acute renal failure, respiratory failure, cerebrovascular accident, deep venous thrombosis, pulmonary embolism, pneumonia, bacteraemia/sepsis, surgical site infection, and postoperative haemorrhage [23]. They were extracted from chart review, and constituted confirmed clinical diagnoses made by consultant-led treating teams.

### 2.5. Statistical Analysis

Demographic, surgical, and outcome information is presented as mean with standard deviation if normally distributed, and otherwise as median with 1st and 3rd quartiles or counts with percentages. Logistic regression was used to assess 30-day mortality and transfusions (“yes”/“no”), negative binomial regression was used for number of transfusion units and normal linear regression was used for POD 1 Hb. Association between time to SAE and T_s_ was assessed with Cox regression. Subjects were censored at death or discharge unless the death was grouped with SAEs. All odds ratios, rate ratios, estimates and hazard ratios are presented as adjusted with 95% confidence intervals. Covariates used for adjustment included age (years), sex, arthroplasty, CCI and eGFR. Admission Hb was additionally used for the day 1 Hb analysis. Figures were plotted with loess smoothing curves. Significance was set at *p* < 0.05. Statistical analyses were programmed using SAS v9.4 (SAS Institute, Cary, NC, USA).

## 3. Results

### 3.1. Trends in Direct Oral Anticoagulant (DOAC) Use

**Figure 1:**
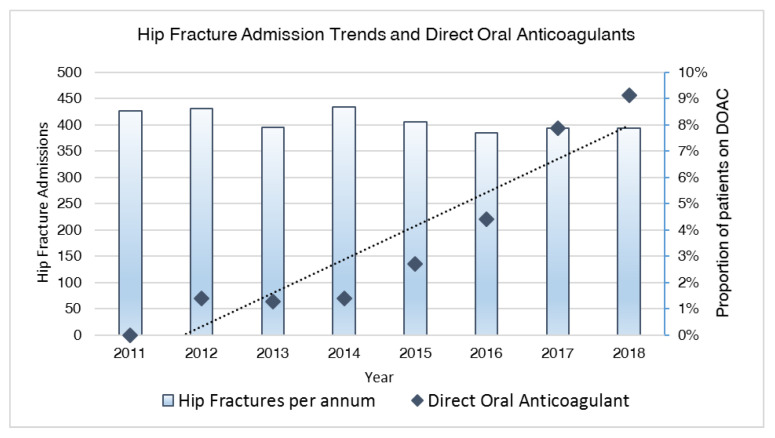
Trends of direct oral anticoagulant (DOAC) use and hip fracture admissions between 2011 and 2018.

A total of 112 subjects were admitted, taking DOACs on admission out of 3264 geriatric hip fractures admitted between 2011 and 2018. Basic demographics are reported (Table 1). A more comprehensive table of comorbidities is in Supplemental Data (Supplementary Table S1).Yearly rates of DOACs use were 0 (0%) in 2011, 6 (1.4%) in 2012, 5 (1.3%) in 2013, 6 (1.4%) in 2014, 11 (2.7%) in 2015, 17 (4.4%) in 2016, 31 (7.9%) in 2017 and 36 (9.1%) in 2018 (Figure 1). 

Thirteen different dosing regimens were identified in the three agents and are not listed. Dabigatran has declined in use since 2012, whilst Apixiban was the most widely used agent toward the year of 2018 (Table 2). Indications for DOAC use were atrial fibrillation in 96 (86%) patients, cerebrovascular disease in 6 (5.4%), deep vein thrombosis in 4 (3.6%), pulmonary embolus in 3 (2.7%), congestive cardiac failure in 2 (1.8%) and ischaemic heart disease in 1 (0.9%).

### 3.2. Surgical Timing

One patient died preoperatively. Of the remaining 111 patients, 29 (26%) had an arthroplasty procedure and 82 (74%) had fixation. Operative details are in Table 3. T_s_ was 2.2 (± 1.0) days and did not change in six years using quantile regression (coeff: −0.02 95% CI: −0.13, 0.09; *p* = 0.690). Surgical delay occurred in 91 (72%) patients (Table 2). Awaiting DOACs to ‘’wash out’’ was cited as the primary cause for delay in 37 (33%) of patients, with optimization of a medical condition not related to anticoagulation the cause for delay in 19 (17%) patients. Lack of access to operating room was the cause of delay in 35 (31%) of patients.

### 3.3. Fracture Pattern and Operation Type

Extracapsular fracture patterns constituted 73 (65%) patients with 39 (35%) intracapsular fractures (Table 1). Fixation was performed in 82 (73%) patients with 30 (27%) arthroplasties (Table 2). Fixation and arthroplasty are compared in Supplementary Table S2. 

### 3.4. PostOperative Course

Median length of stay was 11 days (6.8, 17). Postoperative delirium occurred in 32 (29%) of patients. 

### 3.5. Outcomes

#### 3.5.1. Mortality

The primary outcome of 30-day mortality was evaluated per year: one death (17%) in 2012, 2 (40%) in 2013, 2 (33%) in 2014, 3 (27%) in 2015, 1 (5.9%) in 2016, 4 (13%) in 2017 and 3 (8.3%) in 2018. This equated to a total of 16 (14%) deaths within the cohort. Demographics, perioperative details and postoperative course are included in Supplementary Table S3. 

**Figure 2:**
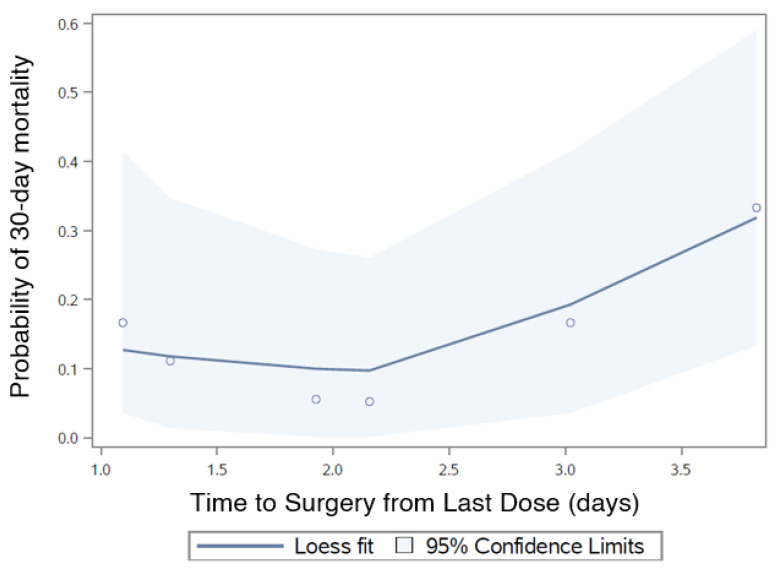
Time to surgery from last dose (T_s_) in relation to proportion of 30-day mortality.

As T_s_ increased by 1 day, a 37% increase in 30-day mortality was seen that did not reach significance (OR: 1.37, 95% CI: 0.80–2.33; *p* = 0.248). Female sex (OR: 0.43, 95% CI: 0.11–1.60; *p* = 0.208) and eGFR (OR: 0.97, 95% CI: 0.94–1.01; *p* = 0.188) did not influence 30-day mortality (Figure 2), but age (OR: 1.12, 95% CI: 0.99–1.27; *p* = 0.064) and CCI (OR: 1.26, 95% CI: 1.00–1.60; *p* = 0.052) was of borderline significance.

#### 3.5.2. Serious Adverse Events

Serious adverse events (SAE) occurred in 25 (22%) patients (Supplementary Table S4). This led to 4 inpatient deaths, leading to a failure to rescue of 16%. The most common were pneumonia in 8 patients (7.1%), followed by acute kidney injury (AKI) in 7 (6.3%) and acute myocardial infarction (AMI) in 3 (2.7%). No patients returned to operating room for evacuation of haematoma or developed deep infections. T_s_ (HR: 1.03, 95% CI: 0.70–1.52; *p* = 0.885), age (HR: 1.01, 95% CI: 0.94–1.09; *p* = 0.738), female sex (HR: 0.61, 95% CI: 0.26–1.44; *p* = 0.257), CCI (HR: 1.10, 95% CI: 0.93–1.30; *p* = 0.274) and eGFR (HR: 0.98, 95% CI: 0.95–1.0; *p* = 0.084) did not show significant influence on time to SAE. 

#### 3.5.3. Transfusion

Transfusion occurred in 30 (27%) patients. Preoperative transfusion occurred in 8 (7.1%) patients, intraoperative transfusion in 6 (5.4%) and postoperative in 22 (20%). Transfusion was not related to T_s_ (OR: 0.72 95% CI: 0.45–1.16; *p* = 0.177), age (OR: 1.02 95% CI: 0.95–1.10; *p* = 0.576), arthroplasty (OR: 1.77 95% CI: 0.58–5.38; *p* = 0.315), CCI (OR: 1.01 95% CI: 0.83–1.24; *p* = 0.908) or renal function (OR: 1.00 95% CI: 0.97–1.02; *p* = 0.745). 

**Figure 3:**
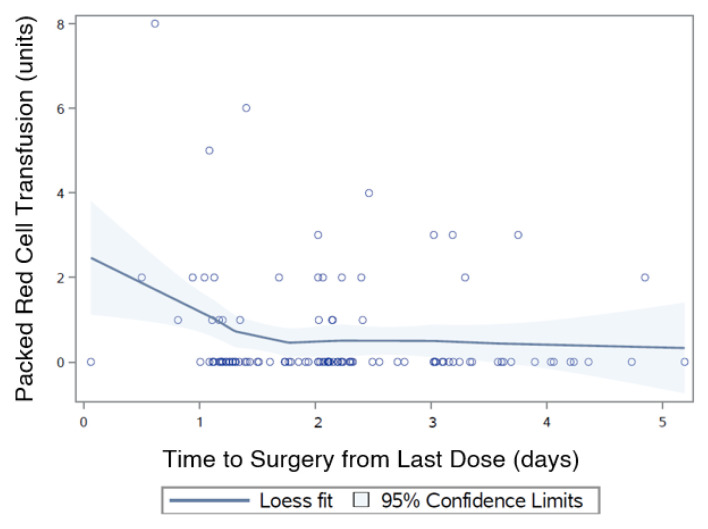
Time to surgery from last dose (T_s_) and number of transfusion units.

Of the patients transfused, 9 patients received 1 unit (8.0%), 11 received 2 units (9.8%), 4 received 3 units (3.6%) and there was 1 patient that (0.9%) received either 4,5,6 or 8 units. As T_s_ increases by 1 day, the average number of transfusion units required decreases by 18%, (OR: 0.82 95%CI 0.54–1.25; *p* = 0.360) but is not statistically significant (Figure 3). 

#### 3.5.4. Haemoglobin Change

**Figure 4:**
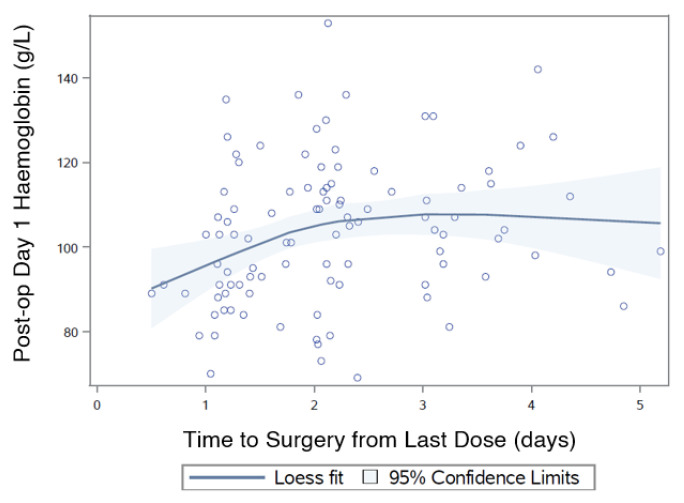
Time to surgery from last dose (T_s_) and postoperative day 1 (POD1) haemoglobin.

Admission Hb was 125 g/L (± 16) and was collected at a median of 2.5 h (1.8,4.0) postinjury and 9.4h (6.1,13) after last dose. Postoperative day (POD) 1 Hb was collected at a median of 22h (18,23) after surgery, with a mean Hb drop from admission of 22 g/L (±14). POD1 Hb when related to T_s_ showed a nonsignificant increase of 2.0 g/L per day (OR: 1.99, 95% CI: −0.59 to 4.57; *p* = 0.129) (Figure 4). Admission Hb significantly predicted POD 1 Hb (OR: 0.63, 95% CI: 0.48–0.79; *p* < 0.001) regardless of transfusion, whilst other variables did not reach significance (Age (OR: −0.18, 95% CI: −0.64 to 0.27; *p* = 0.430); female sex (OR: −1.25, 95% CI:−6.84 to 4.35; *p* = 0.660); no arthroplasty (OR: −5.31, 95% CI:−11.27 to 0.65; *p* = 0.080); CCI (OR: 0.91, 95% CI: −0.32 to 2.13; *p* = 0.145); or eGFR (OR: −0.15, 95% CI: −0.32 to 0.02; *p* = 0.086))

### 3.6. Control Comparison

The age- and sex-matched control group demonstrated a reduced time to operating room from admission compared to the DOAC cohort (1.2 days (±0.7) vs. 1.8 days (±1.3); *p* < 0.001). Less delay was seen for control group patients for lack of surgical time (19 (17%) vs. 35 (31%) *p* = 0.008) and medical optimization (2 (1.8%) vs. 19 (17%); *p* < 0.001). Length of stay (6.9 days (4.2–11) vs. 11 days (6.5–18); *p* < 0.001) and inpatient death (1 (0.9%) vs. 8 (7.1%); *p* = 0.017) was less than in the DOAC group. There was no statistical difference in 30-day mortality (7 (6.3%) vs. 16 (14%); *p* = 0.225) (Supplementary Table S5).

## 4. Discussion

The primary outcome in this study was 30-day mortality, which showed no significant relation to the time to surgery from last dose (T_s_). Mortality figures in this study are similar to the previously reported 30-day mortality in our institution [24]. Age and CCI, whilst commonly being predictive of 30-day mortality [21,24], were of borderline significance in this analysis, likely due to the small sample size. Other studies have demonstrated far lower mortality in groups taking DOACs than matched noncoagulated groups, such as 1.6% (1/63) vs. 8.1% (8/62) [13] and 5.3% (1/19) vs. 16% (12/76) [7]. This perceived mortality benefit from DOACs compared to no anticoagulation is difficult to interpret. The matched control group in our study would suggest that patients prescribed DOACs have more comorbidities, more surgical delay and higher mortality.

The secondary outcome of time to a serious adverse event showed no association with T_s_ or other variables. Transfusion, both as a binary outcome (‘’yes’’/’’no’’) or number of packed cell units transfused, was similarly not influenced by T_s_ or other variables. POD 1 Hb levels were influenced by admission Hb only.

The study has larger numbers than the existing literature (*n* = 7 [6], *n* = 19 [7], *n* = 27 [8], *n* = 28 [9], *n* = 29 [10], *n* = 33 [16], *n* = 47 [11], *n* = 52 [15], *n* = 54 [12], *n* = 63 [13] and *n* = 89 [14]). It also specifically focuses on assessing a dose-response relationship within a cohort taking DOACs; this approach avoids the potential bias of finding a suitable non-DOAC control group and should be considered for future studies. The retrospectively identified non-DOAC treated control groups cannot address the initial decision making of the prescribing physicians. It seems to be that during the introductory phase of the DOACs, patients with better general health were receiving it. We also believe that the definitions of “early versus late” surgical timing (12, 24, 36, 48 or 72 hrs after admission [25]) are arbitrary, and that time is best investigated as a continuum, without assuming linearity.

The existing literature compares hip fracture groups with matched cohorts not taking anticoagulants [7,8,9,11,12,13,14] or groups’ DOACs with other antithrombotics without separate analysis [6,10]. The most common management question asked by clinicians is ‘’what is the safest window to operate?’’. We have attempted to answer this with adjustment for known predictors of mortality, renal function that affects DOAC concentration and surgical type that influences time to surgery. 

Using time to surgery from the last documented DOAC dose (T_s_) is of more clinical interest than time to surgery from admission. As uncertainty exists with DOACs and optimal surgical timing in our institution, last dose was comprehensively recorded in patient records. 

All patients were fully anticoagulated on admission. Recent focus in the fragility fracture research community has been directed at looking at serum drug concentrations and optimal cut-offs for operative intervention. This involves daily testing and delaying surgery until an arbitrary value, such a 30 μg/L, 50 μg/L or 80 μg/L of DOAC concentration, is reached. Thromboelastography (TEG) parameters have been used to accurately assess the presence of DOACs in well, nonsurgical populations without correlation to plasma drug levels [19]. Both may be used as rapid bedside tools in the future; however, uncertainty still remains around whether delay of surgery is advantageous.

Current protocols for preoperative cessation of DOACs are not based on outcomes originating from quality research [18]. They attempt to provide a theoretical reduction in blood loss as per variable pharmacokinetics, but do not consider physiological complexity or time-dependent adverse outcomes. Concerns have been raised with DOACs in patients older than 90 years old regarding the paucity of evidence for this age group [26]. In our cohort, 24 (21%) of patients were aged 90 and above (the eldest 98, lived independently at home with no cognitive impairment). Viktil et al. conducted a pilot study in hip fractures taking DOACs that demonstrated median DOAC half-life was 22 hrs (range 15–60 h range). Operative intervention occurred at a median of 44 hours (22–64) from admission. At time of surgery, 50% of patients were fully anticoagulated reflecting prolonged elimination [27]. Thus, half-lives of DOACs in the elderly are far longer than reported for the normal population [7]. This may help explain why there was no advantage to transfusion or POD1 Hb from delayed surgery in our study. In this regard, DOACs are not dissimilar to clopidogrel: even with a prolonged half-life and no feasible reversal options, surgical delays have no benefit to outcomes [28]. 

Most studies investigating DOACs and hip fractures examine haemoglobin drops and estimated blood loss (EBL). We chose to examine POD 1 Hb, as it is generally the trigger for clinical management decisions. EBL and Hb drops are rarely calculated, and hence do not influence clinical decision making. The curve generated for POD 1 Hb demonstrates there is a nonsignificant upward slope from early surgery. Drop in Hb has been demonstrated to be independent of surgical timing with DOAC-taking hip fractures [13], which echoes our results. The transfusion rate was in the range reported in other studies (18% [13], 37% [8], 38% [15] and 54% [12]), and was not related to surgical timing, as has been previously demonstrated in the United Kingdom [13]. 

Delay to surgery was seen in 72% of patients. Inadequate access to operating room represented delays in 31% of patients; 33% of patients were delayed due to being on DOACs. Comparison with the age- and sex-matched control group showed significantly more delays due to lack of time and medical optimization than patients not taking antithrombotics. This suggests a potential surgical bias to cancel anticoagulated patients or place them in an operative order, so they are not prioritized and hence more prone to cancellation. With no clear consensus in the literature, opinion is still divided on optimal timing. Other studies have concluded that early surgery is not detrimental for DOACs and hip fracture; however, with no internal comparison regarding time as a variable, and no comparison to a ‘’late’’ group, it is hard to support such statements [7]. 

Whilst excellent results have been achieved with mandating early surgery [17], selection bias can exist that registries cannot account for. Recent large database studies have shown no mortality or morbidity benefit for early surgery when accounting for confounding variables [29]. Large meta-analyses have limitations due to big data from administrative databases with absent or poor coding of comorbidities [30]. The definition of ‘’early’’ is mostly divided between 24 and 48 hours between studies [30], which are very different time goals to realistically meet in a busy trauma service. Subsets within the hip fracture cohort may benefit from different surgical timings. Recent evidence from a large cohort has demonstrated sicker patients are not detrimentally affected by surgical delay [31]. In this study, ‘’medical reasons’’ resulted in delay for 17% of patients, which can lead to bias affecting patients with later surgical times. We attempted to account for this with age and CCI adjustment. 

By demonstrating no advantage in surgical timing, a counter-argument can be made for delaying surgery. The trend of 30-day mortality did increase with delay but did not reach significance (Figure 2). Using our figure of a 37% increase in 30-day mortality per delay of 1 day, with a mortality of 14%, the sample size to detect differences for the outcome would be *n* = 2263. 

Limitations of this study are that it is retrospective, single-centered, and spans a time period where there was uncertainty about how to approach patients admitted on DOACs. This initial uncertainty was the driving force for this study, and similar sentiment is likely responsible for the emergence of other literature regarding DOACs and hip fracture in the last 2 years. We have pragmatically studied all of our patients since 2011. Minor outcomes such as wound ooze were not formally analyzed, as it is a particularly difficult minor complication to quantify. Wound outcomes are important to surgeons, even if they are not serious adverse event. The external validity of these results must also be carefully applied: 65% of patients had an extracapsular fracture and 73% received fixationWhilst similar to our results in dual-antiplatelet agents and hip fracture [32], this is higher than the national average of 54% extracapsular fracture [20] and our control cohort. The posthoc calculation suggests this study lacks power. This infers that single institution studies will be unable to answer this question of surgical timing adequately, and large national databases and registries will be needed.

## 5. Conclusions

Timing of surgery did not affect mortality, serious adverse events, transfusions or postoperative day 1 haemoglobin levels. Delaying hip fracture surgery due to DOAC use is not recommended. 

## Figures and Tables

**Table 1:** Descriptive statistics of demographic variables.

Variables		Total (*n* = 112)
Age (years; mean, SD)		84.3 (± 6.1)
Sex (Female; *n*, %)		76 (68%)
Muller fracture pattern (*n*, %)		
	31 A1	30 (27%)
	31 A2	27 (24%)
	31 A3	16 (15%)
	31 B1	10 (8.9%)
	31 B2	6 (5.4%)
	31 B3	23 (21%)
Residence (*n*, %)		
	Home	87 (78%)
	Hostel	1 (0.9%)
	RACF	24 (21%)
Mobility (*n*, %)		
	Independent	49 (43%)
	Walking aid	63 (57%)
Cognition (Impaired; *n*, %)		36 (32%)
CCI (median, Q1–Q3)		1 (0,3)

CCI—Charlson Comorbidity Index; RACF- residential aged care facility.

**Table 2:** Yearly breakdown of DOAC use and surgical delay.

Variable	2012	2013	2014	2015	2016	2017	2018	Total
	(*n* = 6)	(*n* = 5)	(*n* = 6)	(*n* = 11)	(*n* = 17)	(*n* = 31)	(*n* = 36)	(N = 112)
DOAC (type; *n*, %)								
Apixiban	0 (0%)	0 (0%)	2 (33%)	3 (27%)	11 (65%)	21 (68%)	19 (53%)	56 (50%)
Dabigatran	6 (100%)	4 (80%)	1 (17%)	2 (18%)	2 (12%)	2 (6.4%)	1 (2.8%)	18 (16%)
Rivaroxaban	0 (0%)	1 (20%)	3 (50%)	6 (55%)	4 (24%)	8 (26%)	16 (44%)	38 (32%)
Time to Surgery from Last Dose (days; mean, SD)	2.1 (0.7)	3.5 (1.2)	3.0 (1.0)	2.3 (1.1)	1.7 (1.2)	2.0 (1.0)	2.2 (0.8)	2.2 (1.0)
Days Delayed (*n*, %)								
0	2 (33%)	2 (40%)	1 (17%)	3 (27%)	7 (41%)	11 (35%)	5 (14%)	31 (28%)
1	3 (50%)		2 (33%)	5 (45%)	7 (41%)	10 (32%)	23 (64%)	50 (45%)
2	1 (17%)	1 (20%)	3 (50%)	2 (18%)	1 (5.9%)	7 (23%)	6 (17%)	21 (19%)
3+		2 (40%)		1 (9.1%)	2 (12%)	3 (9.7%)	2 (5.6%)	10 (8.1%)
Cause of Delay (reason; *n*,%)								
DOAC	1 (17%)	2 (40%)	1 (17%)	3 (27%)	4 (24%)	9 (29%)	17 (47%)	37 (33%)
Lack of surgical time	1 (17%)	1 (20%)	1 (17%)	3 (27%)	5 (29%)	11 (35%)	13 (36%)	35 (31%)
Medical optimisation	2 (33%)	0 (0%)	3 (50%)	4 (36%)	2 (12%)	2 (6.5%)	6 (17%)	19 (17%)
DOAC- direct oral anticoagulant								

**Table 3:** Descriptive statistics of operative details.

Variable	Total (*n* = 111)
Length of operation (h:mm; mean, SD)	1:30 (± 0:43)
ASA Score (Grade; *n*, %)	
2	11 (9.9%)
3	71 (64%)
4	28 (25%)
5	1 (0.9%)
Anaesthetic (*n*, %)	
General anaesthetic	96 (86%)
Neuraxial anaesthetic	15 (14%)
Operation type (*n*, %)	
Cemented hemiarthroplasty	20 (18%)
Cannulated screws	9 (8.1%)
Dynamic hip screw	4 (3.6%)
Long femoral nail	29 (26%)
Short femoral nail	40 (36%)
Uncemented hemiarthroplasty	6 (5.4%)
Total hip replacement	3 (2.7%)

ASA—American Society of Anesthesiologist; GA—General Anaesthetic.

## Supplementary Materials

The following are available online at https://www.mdpi.com/2077-0383/9/7/2200/s1, Table S1: Additional Descriptive Statistics of Demographic Variables, Table S2: Arthroplasty vs Fixation Demographic Variables and Outcomes, Table S3: Demographics and Outcomes of 30-Day Mortality, Table S4: Serious Adverse Events (SAE) and Mortality, Table S5: Matched Control vs. DOAC Variables and Outcomes.
