# Supplementary material for: Direct Oral Anticoagulants and Timing of Hip Fracture Surgery
Source: J Clin Med. 2020 Jul 12;9(7):2200. doi: 10.3390/jcm9072200 (PMC7408859; doi:10.3390/jcm9072200)
Supplement: Supplementary file 1 [file jcm-09-02200-s001.pdf]

**Supplement Table 1.** Additional descriptive statistics of demographic variables.

| Variables | Total (n=112) |
|---|---|
| Comorbidities (n,%) | |
| Ischaemic Heart Disease | 31 (28%) |
| Congestive Cardiac Failure | 33 (29%) |
| Atrial Fibrillation | 101 (90%) |
| Deep Vein Thrombosis | 7 (6.3%) |
| Pulmonary Embolus | 7 (6.3%) |
| Cerebrovascular Disease | 33 (29%) |
| Hypertension | 68 (61%) |
| Chronic Renal Failure | 15 (13%) |
| Diabetes Mellitus | 15 (13%) |
| Gastrooesophageal Disease | 25 (22%) |
| Chronic Airway Disease | 16 (14%) |
| Malignancy | 11 (9.8%) |
| Comorbidity Scores (median, Q1-Q3) | |
| Nottingham Hip Fracture Score | 5 (4-6) |
| Revised Cardiac Risk Index | 1 (0-2) |

**Supplement Table 2.** Arthroplasty vs fixation demographic variables and outcomes.

| | Arthroplasty (*n* = 29) | Fixation (*n* = 82) | *p* value |
|---|---|---|---|
| Variables | | | |
| Age (years; mean, SD) | 83.3 (±5.5) | 84.7 (±6.3) | 0.294 |
| Sex (Female; *n*, %) | 17 (59%) | 59 (71%) | 0.184 |
| Cognition (Impaired; *n*, %) | 8 (27%) | 27 (33%) | 0.595 |
| CCI (median, Q1-Q3) | 2 (2-2) | 1 (0-3) | 0.498 |
| Time to Surgery from Last Dose (days; mean, SD) | 2.6 (±0.9) | 2.1 (±1.0) | 0.026 |
| Cause of Delay (reason; *n*,%) | 68 (61%) | 68 (61%) | |
| DOAC | 13 (45%) | 24 (29%) | 0.127 |
| Lack of surgical time | 10 (35%) | 25 (30%) | 0.691 |
| Medical optimisation | 5 (17%) | 14 (17%) | 0.984 |
| ASA Score (Grade; *n*, %) | | | 0.689 |
| 2 | 2 (6.9%) | 9 (11%) | |
| 3 | 21 (72%) | 50 (61%) | |
| 4 | 6 (21%) | 22 (27%) | |
| 5 | 0 (0.0%) | 1 (1.2%) | |
| Anaesthetic (*n*, %) | | | 0.561 |
| General anaesthetic | 26 (90%) | 70 (85%) | |
| Neuraxial anaesthetic | 3 (10%) | 12 (15%) | |

| | | | |
|---|---|---|---|
| Transfusion (*n*,%) | | | |
| Preoperative | 2 (6.9%) | 6 (7.3%) | 0.940 |
| Intraoperative | 2 (6.9%) | 4 (4.9%) | 0.679 |
| Postoperative | 3 (10%) | 19 (23%) | 0.136 |
| Haemoglobin (g/dL; mean, SD) | | | |
| Admission | 129 (±14) | 123 (±16) | 0.061 |
| Day 1 postoperative | 112 (±14) | 100 (±17) | **0.003** |
| Drop | 19 (±13) | 22 (±14) | 0.212 |
| Serious Adverse Event (*n*,%) | 6 (21%) | 19 (23%) | 0.783 |
| 30-day Mortality (*n*,%) | 2 (6.9%) | 13 (16%) | 0.225 |

ASA- American Society of Anesthesiologist; CCI- Charlson Comorbidity Index; DOAC- direct oral anticoagulant.

**Supplement Table 3.** Demographics and outcomes of 30-day Mortality.

| **Demographics** | | | **Comorbidity Scores** | | **Preoperative Medical condition** | **Operative Delay and Reasons** | | | | | | **Operative Details** | | **Outcomes** | | | **Mortality** | |
|---|---|---|---|---|---|---|---|---|---|---|---|---|---|---|---|---|---|---|
| **Age (years)** | **Sex** | **Dementia (y/n)** | **CCI** | **NHFS** | | Delay (Days) | Medical Optimisation | Optimisation needed | Lack of Operative Time (y/n) | Delayed due to DOAC (y/n) | Transfusion (y/n) | ASA | Time to OR from admisison (days) | Post op SAE | Delirium (y/n) | Length of Stay (days) | Inpatient Mortality (y/n) | Cause of Death |
| 87 | Male | No | 9 | 6 | Exacerbation of COPD | 0 | No | | No | No | Yes | 3 | 1.3 | | No | 6 | No | Palliated with pneumonia post re-admission (suicide attempt) |
| 89 | Male | No | 4 | 6 | | 3 | No | | No | Yes | No | 3 | 4.4 | | No | 7 | No | Right sided heart failure / AMI |
| 87 | Female | No | 1 | 5 | rAF | 3 | Yes | rAF workup | No | No | No | 4 | 3.9 | LRTI | No | 12 | Yes | Sepsis post-aspiration pneumonia |
| 92 | Female | No | 8 | 6 | | 2 | No | | No | Yes | No | 3 | 3.3 | RF | No | 4 | Yes | Respiratory failure from sepsis |
| 86 | Female | Yes | 3 | 7 | rAF | 2 | Yes | rAF workup | No | No | No | 4 | 4.2 | | No | 6 | No | Not known |
| 89 | Male | No | 2 | 7 | | 3 | No | | No | Yes | Yes | 4 | 4.7 | LRTI | No | 7 | Yes | Sepsis post-pneumonia |
| 90 | Female | No | 3 | 5 | Heart failure | 1 | Yes | Echocardiogram | No | No | No | 3 | 3.5 | | No | 3 | Yes | Cerebrovascular accident |

| | | | | | | | | | | | | | | | | | | |
|---|---|---|---|---|---|---|---|---|---|---|---|---|---|---|---|---|---|---|
| 87 | Female | Yes | 1 | 7 | | 1 | No | | Yes | No | No | 4 | 0.9 | PE | Yes | 5 | No | Respiratory failure from bilateral pulmonary embolus |
| 91 | Male | Yes | 1 | 7 | | 0 | No | | No | No | No | 4 | 0.7 | | Yes | 3 | No | Sepsis and multiorgan failure |
| 97 | Female | No | 5 | 6 | Acute pulmonary odema | 1 | Yes | Diuresis | No | No | No | 4 | 1.8 | LRTI | No | 3 | Yes | Congestive heart failure |
| 86 | Female | No | 7 | 5 | | 0 | No | | No | No | No | 3 | 0.8 | AMI | No | 1 | Yes | Fat embolism |
| 83 | Male | Yes | 2 | 6 | | 0 | No | | No | No | Yes | 3 | 0.7 | AKI | Yes | 10 | No | Aspiration pneumonia |
| 79 | Male | No | 8 | 7 | Anaemic | 2 | Yes | Echocardiogram | No | Yes | Yes | 4 | 2.3 | AKI | No | 3 | No | Haemorrhagic shock |
| 92 | Female | No | 1 | 4 | | 2 | No | | No | Yes | No | 3 | 2.3 | | No | 8 | No | Not known |
| 86 | Male | Yes | 2 | 6 | | N/A | | | | | | | Non-op | | No | 0 | Yes | Pneumonia |
| 94 | Male | Yes | 3 | 9 | NSTEMI | 1 | Yes | Diuresis | No | No | No | 4 | 2.0 | AKI | Yes | 10 | Yes | AMI |

AKI- Acute Kidney Injury; AMI- Acute Myocardial Infarction; CCI-Charlson Comorbidity Index; COPD-Chronic Obstructive Pulmonary Disease; LRTI- Lower Respiratory Tract Infection; Nottingham Hip Fracture Score- NHFS; NSTEMI- Non ST-elevation Myocardial Infarction; OR- Operating Room; PE- pulmonary embolism; rAF- Rapid Atrial Fibrillation; RF- Respiratory Failure; SAE- Serious Adverse Event.

**Supplement Table 4.** Serious Adverse Events (SAE) & Mortality.

| SAE | SAE timing postadmission (days) | Delirium (y/n) | ICU admission (y/n) | Length of Stay (days) | Inpatient Mortality (y/n) | 30-day Mortality (y/n) |
|---|---|---|---|---|---|---|
| LRTI | 2.9 | No | No | 15 | No | No |
| PE | 9.7 | No | Yes | 40 | No | No |
| AKI | 1.0 | No | No | 30 | No | No |
| RF | 16.5 | Yes | No | 31 | No | No |
| LRTI | 2.9 | No | No | 7 | Yes | Yes |
| CVA | 2.9 | No | No | 15 | No | No |
| PE | 3.7 | Yes | No | 5 | No | Yes |
| LRTI | 6.8 | No | No | 10 | No | No |
| LRTI | 0.4 | No | No | 3 | Yes | Yes |
| AMI | 0.9 | No | No | 0 | Yes | Yes |
| AKI | 1.2 | Yes | No | 10 | No | Yes |
| GIH | 6.8 | Yes | No | 22 | No | No |
| LTRI | 12.4 | Yes | No | 19 | No | No |
| LRTI | 2.8 | Yes | No | 10 | No | No |
| CCF | 10.3 | Yes | No | 25 | No | No |
| AKI | 0.9 | No | No | 14 | No | No |
| Sepsis | 14.6 | No | No | 15 | No | No |
| AKI | 1.9 | No | No | 3 | No | Yes |
| LRTI | 2.4 | Yes | No | 8 | No | No |
| LRTI | 11.6 | No | No | 12 | No | No |
| Sepsis | 3.3 | No | No | 24 | No | No |
| AMI | 11.8 | No | No | 14 | No | No |
| AKI / NSTEMI | 0.4 | Yes | No | 10 | Yes | Yes |
| AKI | 1.7 | No | No | 22 | No | No |
| AKI | 1.8 | No | No | 10 | No | No |

AKI- Acute Kidney Injury; AMI- Acute Myocardial Infarction; ICU- Intensive Care Unit; LRTI- Lower Respiratory Tract Infection; NSTEMI- Non ST-elevation Myocardial Infarction; PE- pulmonary embolism; RF- Respiratory Failure; SAE- Serious Adverse Event.

**Supplement Table 5.** Matched control vs DOAC variables and outcomes.

| | | Control ($n$ = 112) | DOAC ($n$ = 112) | $p$ value |
|---|---|---|---|---|
| Variables | | | | |
| | Age (years; mean, SD) | 84.3 (±6.1) | 84.3 (±6.1) | 0.983 |
| | Sex (Female; $n$, %) | 76 (68%) | 76 (68%) | 1.000 |
| | Cognition (Impaired; $n$, %) | 27 (24%) | 36 (32%) | 0.181 |

| | | | |
|---|---|---|---|
| Ischaemic Heart Disease (*n*, %) | 13 (12%) | 31 (28%) | **0.002** |
| Chronic Renal Failure (*n*, %) | 7 (6.2%) | 15 (13%) | 0.072 |
| Diabetes Mellitus (*n*, %) | 5 (4.6%) | 15 (13%) | **0.019** |
| | | | |
| Time to Surgery from Admission (days; mean, SD) | 1.2 (±0.7) | 1.8 (±1.3) | **<0.001** |
| Cause of Delay (reason; *n*,%) | | | |
| DOAC | N/A | 37 (33%) | - |
| Lack of surgical time | 19 (17%) | 35 (31%) | **0.008** |
| Medical optimisation | 2 (1.8%) | 19 (17%) | **<0.001** |
| ASA Score (Grade; *n*, %) | | | 0.096 |
| 2 | 23 (21%) | 11 (9.9%) | |
| 3 | 67 (60%) | 71 (64%) | |
| 4 | 21 (19%) | 28 (25%) | |
| 5 | 0 (0%) | 1 (0.9%) | |
| Anaesthetic (n, %) | | | **<0.001** |
| General anaesthetic | 55 (50%) | 96 (86%) | |
| Neuraxial anaesthetic | 56 (50%) | 15 (14%) | |
| Operation type (*n*, %) | | | **0.038** |
| Cemented hemiarthroplasty | 36 (32%) | 20 (18%) | |
| Cannulated screws | 13 (12%) | 9 (8.1%) | |
| Dynamic hip screw | 5 (4.5%) | 4 (3.6%) | |
| Long femoral nail | 15 (14%) | 29 (26%) | |
| Short femoral nail | 30 (27%) | 40 (36%) | |
| Uncemented hemiarthroplasty | 3 (2.7%) | 6 (5.4%) | |
| Total hip replacement | 9 (8.1%) | 3 (2.7%) | |
| Length of Stay (days; median, Q1–Q3) | 6.9 (4.2-11) | 11 (6.5-18) | **<0.001** |
| Inpatient Mortality (*n*,%) | 1 (0.9%) | 8 (7.1%) | **0.017** |
| 30-day Mortality (*n*,%) | 7 (6.3%) | 16 (14%) | 0.225 |

ASA- American Society of Anesthesiologist; DOAC- direct oral anticoagulant.
